# Model dielectric function for 2D semiconductors including substrate screening

**DOI:** 10.1038/srep39844

**Published:** 2017-01-24

**Authors:** Mads L. Trolle, Thomas G. Pedersen, Valerie Véniard

**Affiliations:** 1 Laboratoire des Solides Irradiés, Ecole polytechnique, CNRS, CEA, Université Paris-Saclay, 91128 Palaiseau, France; 2 European Theoretical Spectroscopy Facility, 91128 Palaiseau, France; 3 Department of Physics and Nanotechnology, Aalborg University, DK-9220 Aalborg Øst, Denmark; 4 Center for Nanostructured Graphene (CNG), DK-9220 Aalborg Øst, Denmark

## Abstract

Dielectric screening of excitons in 2D semiconductors is known to be a highly non-local effect, which in reciprocal space translates to a strong dependence on momentum transfer *q*. We present an analytical model dielectric function, including the full non-linear *q*-dependency, which may be used as an alternative to more numerically taxing *ab initio* screening functions. By verifying the good agreement between excitonic optical properties calculated using our model dielectric function, and those derived from *ab initio* methods, we demonstrate the versatility of this approach. Our test systems include: Monolayer hBN, monolayer MoS_2_, and the surface exciton of a 2 × 1 reconstructed Si(111) surface. Additionally, using our model, we easily take substrate screening effects into account. Hence, we include also a systematic study of the effects of substrate media on the excitonic optical properties of MoS_2_ and hBN.

It is by now recognized that correlated electron-hole pairs, termed excitons, are essential for reliably modelling the optical response of semiconducting materials. The dielectric screening is an essential ingredient in such calculations^1^, and especially so for low-dimensional systems^1^,^2^,^3^,^4^,^5^. Screening in 2D semiconductors, e.g., has recently attracted a tremendous amount of attention. In particular, the question of translating the inherently 3D concept of a dielectric function, calculated *ab initio* in the random phase approximation (RPA) from super-cell geometries, to free-standing 2D materials has been scrutinized in great detail^3^,^4^,^5^. Hence, screening in 2D materials is known to be highly non-local, which in reciprocal space translates into a function depending strongly on momentum transfer ***q***^3^,^4^,^5^,^6^. The study of many-body effects in bulk 3D semiconductors has been aided by the application of model dielectric functions^7^,^8^,^9^,^10^, where the essential *q*-dependence is extrapolated from simple analytical formulas, thus side-stepping numerically taxing RPA response functions. Furthermore, tight-binding (TB) states have recently been applied as a highly efficient basis for constructing exciton wave functions^2^,^11^,^12^,^13^. However, the lack of completeness in such restricted bases typically implies under-screening, necessitating simplified screening models^12^ or *ad hoc* rescaling of RPA results^2^. The latter approach, in particular, is questionable for free-standing 2D materials, where the dielectric screening is known to approach unity^3^ for small *q* - hence a simple scaling of the effective screening by a constant factor leads to the wrong qualitative behaviour for small *q*. Thus, reliable model dielectric screening methodologies are highly attractive for the study of many-body optical properties of 2D systems.

In the present work, we derive an analytical model dielectric screening function for 2D semiconductors, allowing for the description of substrate screening. This model is particularly useful in the context of excitons constructed on the basis of a minimal TB model, but may readily be generalized for *ab initio* methods. To test the applicability of the model presented here, we consider three 2D semiconductors for which rigorous *ab initio* results are available for comparison: Monolayer hBN, monolayer MoS_2_ and the dangling *π*-electron bonds of a 2 × 1 buckling-chain reconstructed^14^ Si(111) surface. The former two cases represent van der Waals bound 2D semiconductors - a family of materials whose optical response are currently being intensively studied^4^,^5^,^12^,^13^,^15^. However, most theoretical work are currently being carried out on free-standing monolayers, neglecting possibly important effects of substrate screening almost always present in experiments. The latter example represents a somewhat different system, for which the substrate cannot be eliminated for obvious reasons. Nonetheless, the surface states of Si(111) 2 × 1 are confined to a *d* ≈ 4 Å surface region, with energies well-isolated from the bulk bands in most of the Brillouin zone^16^,^17^,^18^. Hence, this system has many similarities with a two-band 2D semiconductor, and demonstrates the versatility of the current approach.

In all cases, excitonic optical spectra in excellent agreement with experiments and *ab initio* theory are obtained. In addition, we highlight the effects of semi-infinite substrates on the optical properties. Finally, we estimate the scaling of the exciton binding energy with substrate screening, using both the Bethe-Salpeter equation (BSE) and an effective-mass 2D Wannier model.

## Theory and Methods

We start from a model dielectric function suggested by Cappellini *et al*.^10^, which is valid for 3D semiconductors


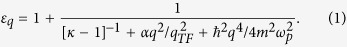


Here, *κ* is the *q* → 0 limit of the static RPA or experimental 3D dielectric function, while the Thomas-Fermi wave vector *q*_*TF*_ and the plasma frequency *ω*_*p*_ both depend (only) on the valence electron density *n(**r***). The Thomas-Fermi wave vector is: 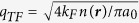, with *k*_*F*_ = [3*π*^2^*n(**r***)]^2/3^ the Fermi wave vector, and *a*_0_ the Bohr radius. Hence, lattice periodic variations in the dielectric function may be included in this way within a kind of local-density approximation^9^,^10^. However, we will not consider local-field effects explicitly in the following, why we take *n* as the average valence electron density, which in ref. 10 was demonstrated to yield results in good agreement with macroscopic bulk dielectric functions. The fitting parameter *α* is used to ensure agreement with RPA calculations, and *α* = 1.5 turns out to be a good fit to most *ab initio* results^10^. In Table 1, we summarize the parameters used for Si, hBN and MoS_2_ (noting that *κ* and *n* are the only free parameters, with *ω*_*p*_, *q*_*TF*_ and *E*_*F*_ following directly from *n*).

Generally, scaling a material from bulk to 2D entails a changing dielectric screening due to at least two distinct phenomena: (i) Reduced dimensions can lead to changes in the electronic structure (due to e.g. confinement effects, surface states, etc.), which, in turn, affect the dielectric response function. (ii) Changing the structural morphology from bulk to a 2D slab geometry obviously entails changes in the electrostatic treatment of interacting charges, e.g. by the introduction of boundary conditions. Keldysh^19^ demonstrated that including the latter, while neglecting the former, for a slab geometry of vanishing thickness described by a local dielectric constant, yielded a non-local effective 2D screening function linear in in-plane momentum transfer *q*. More recently, Latini and co-workers^4^ have provided a model generalized to slab geometries of finite thickness *d* and fully *ab initio* non-local dielectric functions. They demonstrate that the effective 2D screening is nonlinear in general, while they confirm the validity of the linear Keldysh potential for small *qd*. Inspired by the success of the Keldysh model, we treat a 2D slab of finite *d* using a dielectric function local in the confinement *z*-direction. However, recognizing the importance of the in-plane non-locality demonstrated in ref. 4, we retain the in-plane *q*-dependence of the dielectric function, which we model using Eq. 1. This corresponds to the following ansatz for a model dielectric function in real space


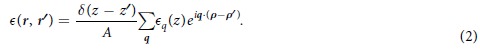


Here *A* indicates the crystal area, ***ρ*** = (*x, y*) is the in-plane coordinate, while 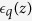 is taken as piecewise constant depending on the medium at *z*.

In the remainder of the Theory and Methods section, we briefly outline the formalism necessary for applying an effective 2D screening function in the BSE framework. This derivation is very similar to that of ref. 4, but is included here for the sake of completeness. We then proceed to model the effective 2D interaction based on the electrostatic boundary value problem of a slab geometry, resulting in an analytic model 2D screening function valid for finite *q* and *d*. Moreover, we discuss the strict 2D *d* → 0 (Keldysh) limit in addition to a small *q* linearisation. Following this, we review the numerical details.

### Screening of 2D excitons in the Bethe-Salpeter equation

When considering excitons in 2D systems, the question of screening becomes a delicate one, and we here adopt an analysis similar to refs 4 and 20. The complication arises when treating the direct screened Coulomb matrix elements entering in the BSE^1^, which couples singly excited states constructed from the ground state Slater determinant by substitution of a valence band *v* with conduction band *c* at ***k*** in momentum space. Upon assuming single-particle wave functions with band index *n* and *k*-vector ***k*** separable on the form Ψ_*n**k***_(***r***) = *ψ*_*n**k***_(***ρ**)g*_*n**k***_(*z*), the screened Coulomb matrix elements may be written as


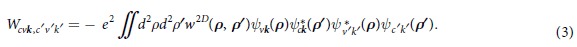


Here, ***ρ*** = (*x, y*) is the periodic in-plane coordinate, while *z* is the coordinate perpendicular to the plane. Further,


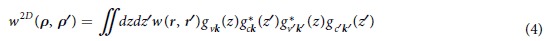


is the 2D screened Coulomb kernel, while 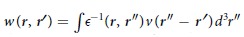 is the usual screened Coulomb interaction, with 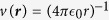. Generally, the screened potential *w*^2*D*^ contains parts which vary on the scale of the unit cell, and is thus represented by the full screened interaction matrix 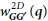 in Fourier space, with ***G*** and ***q*** in-plane reciprocal lattice vectors and Brillouin zone (BZ) wave vectors, respectively. In the present work, we retain only the ***G*** = ***G*****′** = **0** element, which essentially means we apply a Coulomb interaction macroscopically averaged in the plane. This approach is justified by the fact that the rapidly varying components of the integrand in Eq. 3 tend to average out when integrating, and we note that a similar approach has been applied successfully before for van der Waals bound 2D semiconductors^4^.

The *z*-behaviour of the wave functions is dictated predominately by 2D confinement, with *g*_*n**k***_(*z*) fairly rigid. Hence, energy dispersion associated with the *z*-direction is weaker than in-plane dispersion. Thus, we make the ansatz that *g*_*n**k***_(*z*) depends little on band index and wave vector *g*_*n**k***_(*z*) ≈ *g(z*). This strategy has previously yielded good results in ref. 4, where |*g(z*)|^2^ was extracted from *ab initio* calculations or, indeed, taken as a step-function |*g(z*)|^2^ = *d*^−1^Θ(*d*/2 − |*z*|), with *d* the sample thickness. We adopt the latter strategy here, which leads to


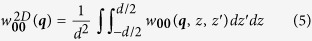


with 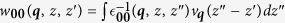, where *v*_***q***_(*z*) is the 2D Fourier transform of *v(**r***).

### Screened Poisson equation

We identify *w*_**00**_(***q**, z, z*′) as the Fourier transform of the electrostatic potential at ***r*** generated by a point charge at ***r*****′**, screened by an effectively in-plane homogeneous electron gas. We accordingly approximate *w*_**00**_ as the solution to the in-plane Fourier transformed screened Poisson equation *φ*_***q***_, using the model dielectric function Eq. 2:


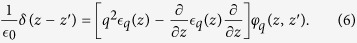


Note, in writing Eq. 6 we have assumed an in-plane isotropic material 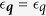, in agreement with previous work^3^,^4^. Furthermore, we assume the dielectric constant *κ* entering 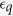 via Eq. 1 to be independent of thickness *d*. This approximation is not equally valid for all materials due to, e.g., quantum confinement affecting the electronic response, or hybridization between the electronic states of the 2D semiconductor and the substrate. However, for the important example of 2D van der Waals stacked semiconductors, such effects are known to be limited due to weak interlayer coupling^21^,^22^.

Solutions to Eq. 6 with the source coordinate *z*′ confined to the ± *d*/2 extent of the sample, and taking 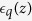 to be piecewise constant


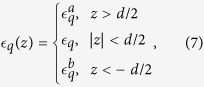


may be found on the form


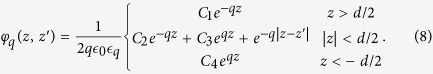


The coefficients *C*_*i*_ are found by solving the appropriate boundary value problem for the potential. Hence, we have access to *φ*_*q*_(*z, z*′), and can perform the integrals in Eq. 5 with *w*_**00**_ ≈ *φ*_*q*_ analytically.

In the case of a free-standing system 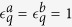 the 2D screened potential becomes


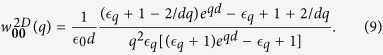


The limit *d* → 0 is well-defined, and yields 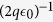 independent of the 2D semiconductor response 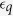. This is a consequence of the dielectric function describing a density response, which must vanish with thickness *d*. However, we may suppose the electric susceptibility *χ*_*q*_ of the vanishingly thin 2D semiconductor to be described by a finite sheet electric susceptibility 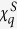, and since 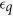 is a macroscopic quantity, we may write 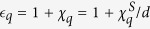. This expression yields the well-known^3^,^4^,^19^ strict 2D *d* → 0 limit of a free-standing film 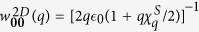. Note, the definition of the 2D susceptibility 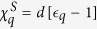 corresponds to requiring the sheet of infinitesimal thickness to posses the same *z*-averaged polarization as the corresponding slab of finite width *d*, similarly to ref. 3.

### 2D model dielectric function

We might suppose an effective 2D dielectric screening function 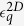, which satisfies


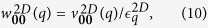


where 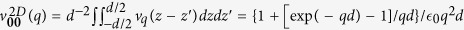 is the 2D bare interaction also found by setting 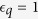 in Eq. 9. Hence, dividing 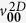 by 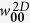 from Eq. 9 we may calculate an effective 2D model dielectric function for a free-standing film of arbitrary thickness *d*, using the expression (with *β* = *qd*):


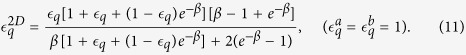


Taking 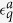 and 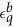 different from unity allows for the introduction of substrate media, and this complication enters naturally through the boundary value conditions for ∂*φ*_*q*_/∂*z*. Analytical coefficients *C*_*i*_ may still be found, and an analytical expression for the model 2D effective screening function is


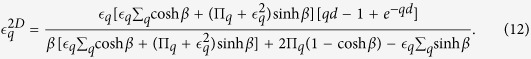


Here, we have used the abbreviations 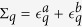, and 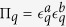.

It has become quite popular to apply a linearised 2D screening valid for small *q*, and excitonic properties using this linearisation are often in surprisingly good agreement with results generated using the full *q*-dependence^3^,^4^. In this spirit, we report also the *q*-linearised version of Eq. 12, valid for small *q*


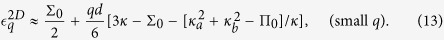


Note that while the free-standing 2D case approaches unity for *q* → 0, the effective screening of the supported 2D system approaches the average substrate screening Σ_0_/2. We may also take the strict 2D limit *d* → 0, which yields simply


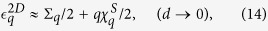


in full agreement with previously published results^3^,^19^. If we further restrict ourselves to the small *q* limit where 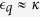, we arrive at the Keldysh result^19^


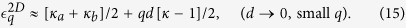


Note, in the *d* → 0 limit the bare 2D interaction for use with Eqs. 14 and 15 reduces to 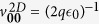. Thus, with Eq. 1, Eqs 11, 12, 13, 14, 15 provide a fully analytical 2D screening model, which takes the dielectric constant *κ* and the average electron density *n* for substrate and film as input.

**Figure 1:**
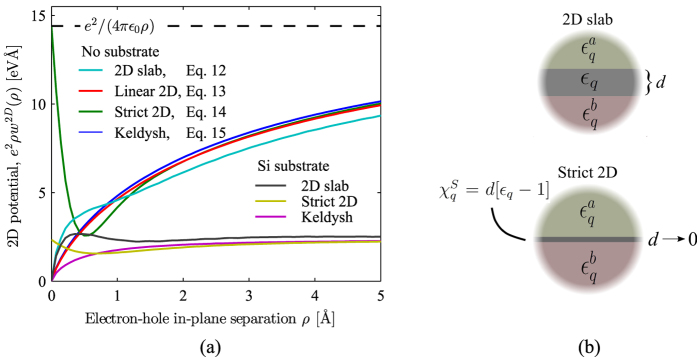
(**a**) Real space electron-hole interaction potentials. The results denoted “2D slab”, “Linear 2D”, “Strict 2D” and “Keldysh” are screened using Eqs 12, 13, 14 and 15, respectively. (**b**) Schematic representation of the slab geometry and its strict 2D limit.

In Fig. 1(a), we plot the 2D potential for an hBN film (*d* = 3.2 Å) in real space, treating it either as a 2D slab (Equation 12), using the *q*-linearised screening (Equation 13), taking the strict 2D limit with finite *q* (Equation 14), or taking the Keldysh limit of small *q* and *d* → 0 (Equation 15). These results are simply the Fourier transform of the screened potential in *q*-space. We compare potentials for a free-standing film to results for a film deposited on a Si substrate. In the latter case, results derived from the *q*-linearised screening (not shown) become un-physical due to the negative slope of Eq. 13, which would imply negative screening for large *q*. The potentials for a free-standing film are almost identical to those published in ref. 4 (with the exception of the strict 2D result valid for finite *q* and *d* → 0) upon noting that we scale our potentials by *ρ*, which makes the differences between the models more apparent. The largest disagreement between the various models is noticed for short-range interaction. These differences may be traced back to the various simplifications affecting the screening functions at large *q*, which in real space translate to differing short-range behaviour. For example, the strict 2D result Eq. 14 becomes unscreened for *q* → ∞, which causes the short-range part of the potential to approach a simple *ρ*^−1^ Coulomb interaction screened by the average of the substrate dielectric constants for *ρ* → 0. This is in marked contrast to the *q*-linear results Eqs 13 and 15, which tend to over-screen the large *q*/small *ρ* part of the interaction. The full 2D slab model screened by Eq. 12 tends to an intermediate regime, with the short range screening enhanced relative to the strict 2D model Eq. 14, but weaker than for the *q*-linear models Eqs 13 and 15. Hence, care must be taken when using linear screening models in the context of strongly bound and localized Frenkel-like excitons, which are particularly sensitive to short range interaction.

In Eq. 6, we might have assumed an anisotropic screening by taking the dielectric constant on a tensorial form 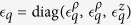. This complication would lead to an effective 2D screening identical to Eq. 12, but with the substitutions 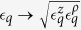, 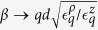, and an overall factor of 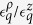. Consequently, the *q*-linearised result would be identical to Eq. 13, with the exception that the first and second *κ* should be substituted by the in-plane and out-of-plane dielectric constants, respectively. However, the strict 2D limit Eq. 14 would contain only the in-plane dielectric constant. In the following sections, we neglect this complication and demonstrate that this is sufficient to achieve agreement with *ab initio* results.

### Computational details

We formulate the exciton problem in a TB basis using a methodology described previously^12^, with the exception that the screened Coulomb kernel 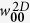 is used instead of the smeared Coulomb potential applied in refs 12, 13 and 15. All TB band structures are parametrized to yield band gaps ensuring the correct spectral positions of the fundamental exciton features taken from experiments, hence, we do not perform an explicit GW calculation in the present work. Instead, we take the TB bands as a model for the corresponding GW energies, c.f. Table 2. Spin-orbit coupling is included only for MoS_2_. For MoS_2_ and Si we apply an *sp*^3^*d*^5^ basis as described in refs 12 and 23, respectively. The Si (111) surface is described using a slab geometry with one face H-terminated, leaving only a single set of surface states in the band gap. Note that in order to achieve the band gap listed in Table 2, the onsite energies of the top buckled surface atoms were adjusted by ± 15%. This is necessary to take into account charge redistribution on the reconstructed atoms and the reduced screening of the surface region (suggesting a larger GW correction compared to bulk for states localized there), even though the TB parameters^23^ were fitted to a GW band structure. The MoS_2_ parameters are fitted to a DFT band structure, hence, we apply a scissor correction in that case. For hBN, a *π*-electron *p*_*z*_ basis is sufficient. We apply the same model as in ref. 13, with the exception that we use onsite energies of ± 2.4 eV for B and N sites, respectively, and a scissor correction of 3.2 eV.

In all cases, we apply dense *k*-grids in excess of 100 × 100 *k*-points using a recursive Lanczos/Haydock scheme^12^, converging the exciton binding energies to the meV level and smearing out the contribution from the individual *k*-points in the continuum part of the spectrum. For hBN and Si (111), we include the valence and conduction band closest to the Fermi level, while for MoS_2_ we include the four lowest conduction bands and four highest valence bands. Where nothing else is noted, we apply a phenomenological broadening of 30 meV.

**Figure 2:**
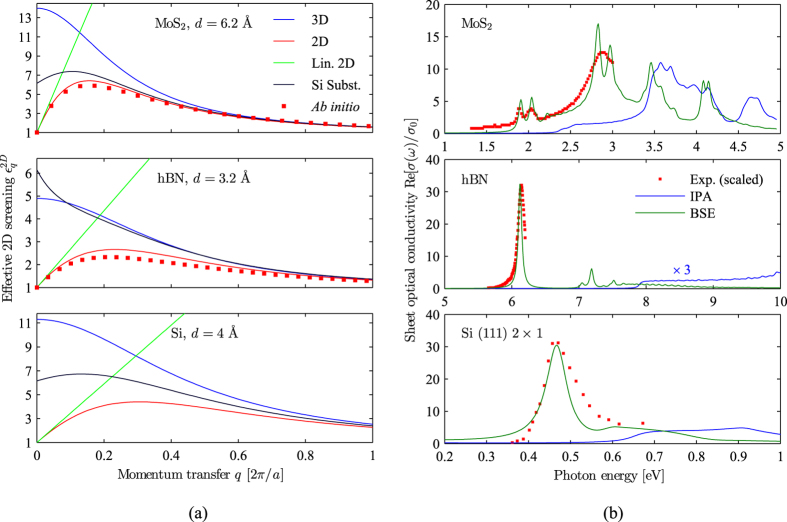
(**a**) The effective screening for three different 2D semiconductors. We include results with the three materials deposited on a Si substrate and *ab initio* results from ref. 5 for comparison. (**b**) Optical sheet conductivity for the three materials considered, calculated both including excitons (BSE), and neglecting many-body effects in the independent particle approximation (IPA). For MoS_2_ and Si(111), we compare with experimental differential reflectance spectra, which are directly proportional to the calculated sheet conductivities. These experimental results are from refs 24 and 27, respectively. For hBN, we compare with the absorption spectra of monolayer hBN flakes suspended in solution from ref. 26. Note, all experimental spectra are scaled for comparison with theory.

We compare exciton energies *E*_*b*_ generated using our BSE approach with results calculated using the well-known and much simpler 2D Wannier exciton model (see e.g. refs 4 and 6): 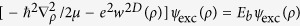. We apply a variational ansatz for the exciton wave function 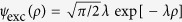, and determine the parameter *λ* by minimizing the energy *E*_*b*_. The reduced electron-hole mass *μ* is extracted from the near-parabolic dispersion of the TB bands near the K-points of the Brillouin zone, and is 0.24*m*_*e*_ for MoS_2_ and 0.36*m*_*e*_ for hBN, with *m*_*e*_ the free-electron mass. The screened potential in real space *w*^2*D*^(*ρ*) is taken as the inverse Fourier transform of Eq. 10 either (i) keeping *d* finite and using Eq. 12 for screening, or (ii) taking the *d* → 0 limit and applying Eq. 15 for screening. The latter case results in the Keldysh potential^19^ 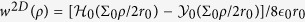. The screening length is 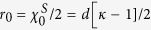, while 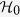 and 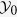 indicate the Struve function and Bessel function of the second kind, respectively. Note, in deriving the Keldysh potential, we apply the strict *d* → 0 limit Eq. 14 for the 2D dielectric function, instead of the *q*-linearised expression for finite *d* Eq. 13, which would also yield a Keldysh-type potential. In fact, the latter approach was shown in ref. 4 to yield good results in the absence of substrate. However, for substrate screening *κ*_*a*_ > *κ*, the linearised dielectric function in Eq. 13 may have a negative slope in *q*-space, as briefly mentioned before, and which can be verified from the hBN results in Fig. 2(a). This leads to un-physical results due to negative screening at large *q*.

## Results and Discussion

We consider initially the effective 2D screening displayed in Fig. 2(a), and note the good overall quantitative agreement between results derived using Eq. 12, and those calculated using more complicated quantum-based approaches^4^,^5^. In fact, the overall agreement is surprising given the simplicity of Eqs 1 and 12 when compared to the full numerical problem of calculating RPA dielectric functions, correctly taking local-field effects into account, avoiding spurious Coulomb interaction between artifically repeated images, etc. Hence, the results of ref. 4 constitute a more numerically taxing problem, albeit with the advantages of being derived from *ab initio* theory. Equation 12, on the other hand, represents a model expression which can be used to extrapolate results very similar to 2D RPA calculations, in the same way the original 3D expression Eq. 1 is used for bulk semiconductors^9^,^10^. The significant qualitative differences between free-standing 2D and 3D screening functions for small *q* have been discussed exhaustively before^3^,^4^, however, the effects of including screening from a supporting substrate (chosen as Si in Fig. 2(a)) are clearly seen to impact the small-*q* range, which is important for excitonic optical properties^5^.

In Fig. 2(b), we include optical sheet conductivities in units of 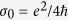, calculated both in the independent particle approximation (IPA) and including excitons through the BSE screened using the full 2D slab model Eq. 12. All excitonic spectra are in excellent agreement with previously published theoretical work and experimental findings.

In Table 2, we compare the magnitude of the exciton binding energies |*E*_*b*_| calculated here with *ab initio* results from other sources. We note a very good quantitative agreement, which suggests a high degree of transferability of the model dielectric function applied in this work. The excitonic optical spectrum of MoS_2_ has been measured experimentally in e.g. refs 21 and 24, and our model reproduces the pronounced exciton peak structure nicely. One exception is the splitting of the peak near 3 eV, which is not observed experimentally. This discrepancy may be explained by electron-phonon interaction, essentially resulting in a frequency dependent broadening^25^. Similarly, the hBN spectrum is dominated by a single intense peak, in good agreement with experiments^26^. A surface exciton peak has also been observed^27^ for Si (111) 2 × 1, with a line shape very similar to Fig. 2(b) and peak position at 0.47 eV.

Having established the reliability of the model dielectric function, we proceed to study the effects of various substrates upon noting that the present model can be expected to work only for cases where the states of the 2D semiconductor hybridizes weakly with the substrate. We consider the case of hBN and MoS_2_ supported by various materials, hence, one half-plane is vacuum while the other is occupied by a material of dielectric constant 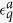 modelled using Eq. 1. However, top-substrates can easily be incorporated, allowing for the simulation of embedded 2D materials. Also, we find the *q*-dependence of 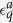 and 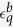 to have negligible influence on the effective 2D screening. Hence, although this complication is included in the present paper, it may safely be neglected in future work, setting 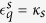.

**Figure 3:**
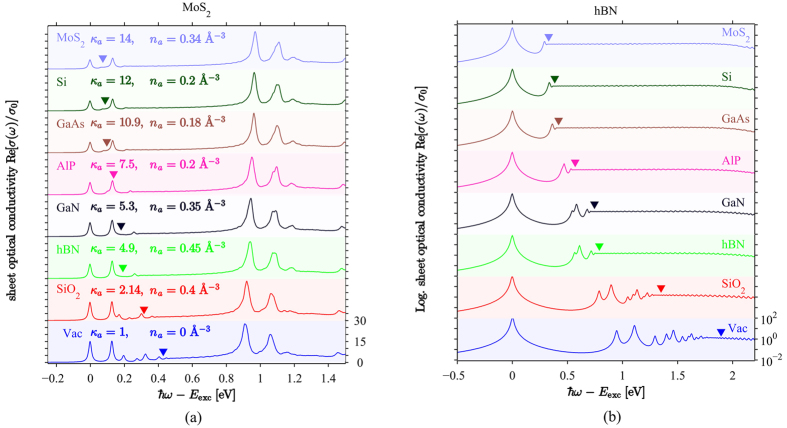
Excitonic optical response of MoS_2_ (**a**) and monolayer hBN (**b**) deposited on the various substrates indicated on the figures. *κ*_*a*_ (in-plane) values are from ref. 4 (MoS_2_, hBN), ref. 29 (Si, GaAs, AlP), ref. 30 (GaN) and ref. 31 (SiO_2_). On the figure, we also indicate the average valence charge densities *n*_*a*_ used to model the *q*-dependence of the substrate dielectric function in Eq. 1. Note, the energy axis is displaced by the exciton binding energy. Hence, the locations of the band gaps on the displaced energy axis correspond to exciton binding energies, and are indicated by the triangles. To resolve the exciton fine-structure, the *k*-point sampling is increased to 200 × 200 for hBN and 150 × 150 for MoS_2_. Additionally, the phenomenological broadening is reduced to 10 meV. Note, the results for hBN are displayed using a logarithmic *y*-axis due to the dominating main absorption peak, and the *y*-limits of all figures are identical to the results for vacuum.

**Figure 4:**
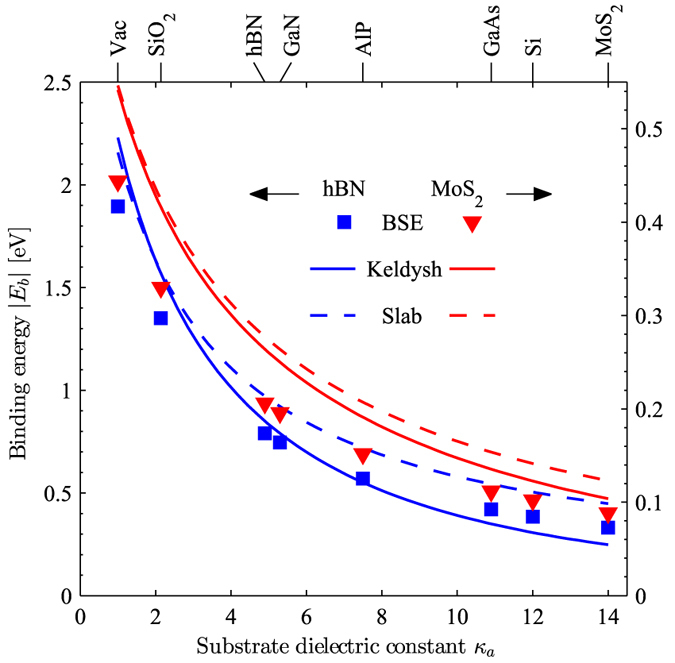
Binding energies of the lowest exciton for hBN and MoS_2_ deposited on various substrates. Squares and triangles indicate results extracted from the BSE, the dashed lines are the 2D Wannier model results for a slab of finite thickness *d*, while the full lines are the strict 2D limit of the same.

In Fig. 3, we display optical spectra for various substrates. In all cases, we observe a dramatic reduction of the exciton binding energy |*E*_*b*_| with increasing substrate screening *κ*_*a*_, as may also be verified from Fig. 4. However, we expect the GW gap *E*_*g*_ to decrease by an almost identical value, similarly to what is typically observed from *ab initio* calculations, such as ref. 28. Hence, we opt to displace the photon energy axis by the energy of the first exciton peak *E*_exc_ = *E*_*g*_ − |*E*_*b*_|. For hBN, we clearly observe the exciton Rydberg series in the energy interval *E*_exc_ to *E*_*g*_. Although the overall spectral structure is conserved with changing substrate, i.e. the dominating main absorption peak is roughly one order of magnitude larger than subsequent peaks, we see that the exciton fine-structure is highly dependent on substrate screening, and almost completely quenched for *κ*_*a*_ > 10.

For MoS_2_, the main effect of increasing substrate screening is to reduce the intensity of the main exciton features, and to blue-shift the peaks near 1 eV on the displaced energy axis. Hence, the most intense peak at 0.9 eV for free-standing MoS_2_ is found at 0.97 eV for the case of a Si substrate. These are attributes which may be measurable experimentally. Furthermore, for substrates with smaller dielectric constants *κ*_*a*_ < 3, an exciton fine-structure is observed in the energy range between *E*_exc_ and *E*_*g*_. However, with increasing substrate screening, the Rydberg series is compressed towards the band gap, giving rise to a slight bulge in the optical spectrum just below *E*_*g*_. Hence, for a GaAs substrate, this bulge is seen as a shoulder on the low-energy side of the second exciton peak. Interestingly, for moderate screening *κ*_*a*_ > 2, the Rydberg series arising from the exciton of second-lowest energy gives rise to a bulge in the continuum part of the spectrum. For a GaN substrate, this is observed near 0.2 eV on the displaced energy axis.

In Fig. 4, we compare BSE exciton binding energies (squares and triangles) to results generated using the much simpler 2D Wannier model, which describes either an exciton confined to a homogeneous slab of finite width screened using Eq. 12 (full lines), or a strict 2D exciton screened using the Keldysh model Eq. 15 (dashed lines). All three models display the same qualitative behaviour, i.e. a decreasing binding energy with increasing substrate screening. However, a few differences are immediately apparent. For a free-standing film, the slab and Keldysh models are in fair agreement in overestimating the exciton binding energies, compared to the BSE. This trend is similar to what has been reported before^4^. We have tested our BSE approach upon substituting the TB energies with parabolic bands extrapolated from the nearest K-point of the Brillouin zone, and we find the resulting binding energies to agree well with the 2D Wannier results. Hence, we attribute the overestimation of the binding energies in the slab and Keldysh exciton models to the effective-mass approximation inherent to these. Upon increasing the substrate screening, binding energies predicted using the strict 2D Keldysh potential decrease more rapidly than predicted by both the finite slab model and the BSE. This trend is especially pronounced when the substrate dielectric constant *κ*_*a*_ becomes larger than the 2D semiconductor dielectric constant *κ*, which is most pronounced for the case of hBN with *κ* = 4.9. Hence, we interpret this as a slight over-estimation of substrate screening in the Keldysh model, as might be expected when taking the 2D limit, thereby reducing the exciton-substrate effective distance to zero.

## Conclusion

In conclusion, we have demonstrated how an analytical model dielectric function may be applied as a reliable tool for predicting the excitonic optical properties of 2D semiconductors. Thus, the calculation of numerically taxing RPA response functions, which is a typical ingredient in exciton calculations, may be sidestepped. This approach was demonstrated to be particularly effective in the framework of tight-binding, where under-screening due to incomplete basis sets can yield unreliable exciton binding energies. We predict substrate screening to generally have a large impact on the exciton binding energy of supported 2D semiconductors. The exciton Rydberg series, in particular, is strongly suppressed with increasing substrate screening. The popular 2D Wannier model is demonstrated to yield results in qualitative agreement with the BSE approach.

## Additional Information

**How to cite this article**: Trolle, M. L. *et al*. Model dielectric function for 2D semiconductors including substrate screening. *Sci. Rep.* **7**, 39844; doi: 10.1038/srep39844 (2017).

**Publisher's note:** Springer Nature remains neutral with regard to jurisdictional claims in published maps and institutional affiliations.

## Figures and Tables

**Table 1:** Parameters applied in Eq. 1.

	*n* [Å^−3^]	*κ*	*ħω*_*p*_ [eV]	*q*_*TF*_ [Å^−1^]	*E_F_* [eV]
hBN	0.45	4.9^*a*^	25.34	2.40	12.47
MoS_2_	0.34	14.0^*a*^	21.60	2.28	17.71
Si	0.20	11.3^*b*^	16.60	2.09	21.91

Note, *E*_*F*_, *ω*_*p*_, and *q*_*TF*_ depend only on the average electron density *n*. The superscripts *a* and *b* refer to refs 4 and 29, respectively.

**Table 2:** Comparison of exciton binding energies |*E*_*b*_| and GW band gaps.

	MoS_2_	hBN	Si(111)
**Binding energy**			
Present	0.44	1.90	0.20
*Ab initio*	0.15^22^, 0.43^4^, 0.65^25^	1.8^32^, 2.05^4^, 2.1^20^	0.26^18^
**GW and TB gaps**			
Present	2.42	8.00	0.66
*Ab initio*	2.48^33^, 2.7^25^	7.9^32^, 7.4^20^	0.62^17^, 0.69^18^

All values are in eV. Note, only exciton binding energies are actually calculated in the present work. Appropriate TB band gaps are ensured by using TB parameters which ensure agreement between experimental and theoretical exciton features. Superscripts indicate references.
